# An upstream open reading frame regulates vasculogenic mimicry of glioma via ZNRD1‐AS1/miR‐499a‐5p/ELF1/EMI1 pathway

**DOI:** 10.1111/jcmm.15217

**Published:** 2020-05-05

**Authors:** Mo Wang, Chunqing Yang, Xiaobai Liu, Jian Zheng, Yixue Xue, Xuelei Ruan, Shuyuan Shen, Di Wang, Zhen Li, Heng Cai, Yunhui Liu

**Affiliations:** ^1^ Department of Neurosurgery Shengjing Hospital of China Medical University Shenyang China; ^2^ Liaoning Clinical Medical Research Center in Nervous System Disease Shenyang China; ^3^ Key Laboratory of Neuro‐oncology in Liaoning Province Shenyang China; ^4^ Department of Neurobiology School of Life Sciences China Medical University Shenyang China; ^5^ Key Laboratory of Cell Biology Ministry of Public Health of China China Medical University Shenyang China; ^6^ Key Laboratory of Medical Cell Biology Ministry of Education of China China Medical University Shenyang China

**Keywords:** competitive endogenous RNA, glioma, nonsense‐mediated RNA decay, upstream open reading frame, vasculogenic mimicry, ZNRD1‐AS1

## Abstract

Increasing evidence has suggested that gliomas can supply blood through vasculogenic mimicry. In this study, the expression and function of ZNRD1‐AS1‐144aa‐uORF (144aa‐uORF) and some non‐coding RNAs in gliomas were assessed. Real‐time quantitative PCR or Western blot was used to discover the expression of 144aa‐uORF, ZNRD1‐AS1, miR‐499a‐5p, ELF1 and EMI1 in gliomas. In addition, RIP and RNA pull‐down assays were applied to explore the interrelationship between 144aa‐uORF and ZNRD1‐AS1. The role of the 144aa‐uORF\ZNRD1‐AS1\miR‐499a‐5p\ELF1\EMI1 axis in vasculogenic mimicry formation of gliomas was analysed. This study illustrates the reduced expression of the 144aa‐uORF in glioma tissues and cells. Up‐regulation of 144aa‐uORF inhibits proliferation, migration, invasion and vasculogenic mimicry formation within glioma cells. The up‐regulated 144aa‐uORF can increase the degradation of ZNRD1‐AS1 through the nonsense‐mediated RNA decay (NMD) pathway. Knockdown of ZNRD1‐AS1 inhibits vasculogenic mimicry in glioma cells by modulating miR‐499a‐5p. At the same time, miR‐499a‐5p is down‐regulated and has a tumour‐suppressive effect in gliomas. In addition, ZNRD1‐AS1 serves as a competitive endogenous RNA (ceRNA) and regulates the expression of ELF1 by binding to miR‐499a‐5p. Notably, ELF1 binds to the promoter region of EMI1 and up‐regulates EMI1 expression, while simultaneously promoting vasculogenic mimicry in glioma cells. This study suggests that the 144aa‐uORF\ZNRD1‐AS1\miR‐499a‐5p\ELF1\EMI1 axis takes key part in regulating the formation of vasculogenic mimicry in gliomas and may provide a potential target for glioma treatment.

## 1 INTRODUCTION

Gliomas occupy 80% of the malignant primary tumours of the human central nervous system, and their prognosis is extremely poor.^1^ Glioma is an angiogenic solid tumour; its malignant progression relies on the formation of blood vessels in tumour tissue.^2^ Although some advances have been made in anti‐angiogenic therapy applied to glioma,^3^ the effect is still insufficient to be relied upon as a primary therapy.^4^ One of the limiting factors of anti‐angiogenesis is related to the generation of vasculogenic mimicry (VM) in glioma cells. The generation of VM is correlated with the pathological grade of a given glioma and promotes the proliferation, migration and invasion in glioma cells.^5^ Therefore, a new mechanism to reveal the VM of glioma cells contributes to the study of the developmental mechanisms of glioma and may contribute new ideas to the remedy for glioma.

At the translational level, the regulation of gene expression depends primarily on the specific structure and function of the 5′ and 3′ untranslated regions (UTR) of mRNA.^6^ Upstream open reading frames (uORFs), commonly found in the 5′UTR of eukaryotic genes, are of importance in the initiation of translation.^7^ Studies have reported that uORFs are present in 44%‐49% of human genes and uORFs in a gene activate nonsense‐mediated RNA decay (NMD), which promotes the degradation of the gene.^8^, ^9^ In this study, an ORF finder was used to predict the presence of a uORF. This was accomplished by encoding a length of 144 amino acids in the 5′UTR of ZNRD1‐AS1. The main point of our study was to explore the regulatory mechanism and effect of ZNRD1‐AS1‐144aa‐uORF (144aa‐uORF) on VM in glioma.

A long non‐coding RNA (lncRNA) is an RNA >200 nucleotides in length and participates in the development of tumours such as glioma.^10^ ZNRD1 (Zinc ribbon domain‐containing 1) is located on chromosome 6p21.3. In its upstream region, there is a lncRNA gene that encodes the ZNRD1 antisense RNA, ZNRD1‐AS1, which is located on chromosome 6p22.1. Emerging evidence has confirmed that ZNRD1‐AS1 expression is great in lung cancer tissues, and that ZNRD1‐AS1 and its functional Cis‐eQTL promote lung cancer.^11^ Presently, there is no report on the expression of ZNRD1‐AS1 in glioma and its involvement in functional regulation.

MicroRNAs (miRNAs/miRs), which are comprised of 18‐25 nucleotides, are a kind of small non‐coding RNA molecules. As regulators of gene expression, miRNAs cause mRNA degradation and/or inhibit protein translation by binding to the 3′UTR of the target gene directly.^12^, ^13^ miR‐449a‐5p is located on the chromosome 5q11.2. Recent studies have reported that miR‐499a‐5p acted a tumour‐suppressive role in gliomas, inhibited migration and invasion of glioma cells and promoted apoptosis.^14^ There is a class of RNA molecules that regulate the action of miRNAs in RNA regulatory networks. For instance, transcripts, such as those from lncRNA, may regulate each other's expression levels and functions through competitive binding of miRNA recognition elements to miRNA. These RNA molecules are called competitive endogenous RNA (ceRNA).^15^ Therefore, whether ZNRD1‐AS1, which is abnormally expressed in glioma cells, can act as a ceRNA to regulate glioma VM, has attracted the attention of the authors.

E74 like ETS transcription factor 1 (ELF1), which belongs to the ETS family, is located in the 13q13.3‐13q14.11 region. Its encoded protein is mainly expressed in lymphocytes, and it acts as an enhancer that regulates the transcriptional expression of various genes as well as the development of breast cancer, melanoma and cervical cancer.^16^, ^17^, ^18^ ELF1 expression is abnormal in many tumours. For example, in endometrial and ovarian cancer, ELF1 is vastly expressed and has a positive correlation with pathological grades and poor prognosis.^19^, ^20^

Early mitotic inhibitor‐1 (EMI1), also named as FBXO5 (F‐box only protein 5), is a key cell cycle regulator. Studies have proposed that, in hepatocellular carcinoma tissues and cells, EMI1 was excessive, promoted cells proliferation and was negatively correlated with the patients' prognosis.^21^ In breast cancer tissues and cells, expression of EMI1 was increased and was positively associated with poor prognoses.^22^, ^23^ It was highly expressed in ovarian clear cell carcinoma tissues and participated in the regulation of its development and progression.^24^ At present, the role of ELF1 and EMI1 in VM formation has many unknowns.

Our study first identified the endogenous expression of ZNRD1‐AS1‐144aa‐uORF, ZNRD1‐AS1, miR‐499a‐5p, ELF1 and EMI1 in tissues and cells of glioma and further studied the above‐mentioned intermolecular regulatory relationship, and the role and mechanism of biological behaviours such as VM formation in gliomas. The research aims to provide a new basis for the occurrence and development of glioma and also point out fresh targets for molecular‐targeted glioma therapy.

## 2 MATERIALS AND METHODS

### 2.1 Human tissue samples

Human glioma tissues and normal brain specimens were taken from at Shengjing Hospital of China Medical University. We received informed consent from the participating patients, and this experiment was approved by the Ethics Committee of Shengjing Hospital of China Medical University. Specimens were cryopreserved in liquid nitrogen instantly after surgery. According to the WHO classification of tumours in the central nervous system (2007) by neuropathologists, the obtained glioma tissues were classified into low‐grade glioma (WHO I‐II: n = 12) and high‐grade glioma (WHO III‐IV: n = 12). Negative controls were those from normal brain tissue obtained from patients with traumatic brain surgery without any significant brain and brain diseases.

### 2.2 Cell culture

The Cell Resource Center of the Shanghai Institute of Biological Sciences provides paid for human glioma cell lines (U87 and U251) and human embryonic kidney cell line (HEK293T). About 10% foetal bovine serum (FBS, Gibco) was added to Dulbecco's modified Eagle medium (DMEM) for culture of U87 and HEK293T cells, and 10% foetal bovine serum was added to DMEM/F12 medium for culture of U251 cells. The humidified incubator was set to 37°C with 5% CO2 and was applied to cell culture.

### 2.3 RNA extraction and quantitative real‐time PCR (qRT‐PCR)

Total RNA of brain tissue samples and HA, U87 and U251 cells were extracted by TRIzol reagent (Life Technologies Corporation). The Nanodrop spectrophotometer (ND‐100, Thermo) was adjusted to a ratio of 260/280 nm for measuring RNA concentration. Primers for ZNRD1‐AS1, ELF1, EMI1, GAPDH, miR‐499a‐5p and U6 were designed and synthesized by Thermo Fisher. qRT‐PCR detection of ZNRD1‐AS1, ELF1, EMI1 and GAPDH was achieved utilizing a one‐step SYBR PrimeScript RT‐PCR kit (Takara Bio, Inc). cDNA from miRNA was synthesized by utilizing TaqMan miRNA Reverse Transcription kit (Applied Biosystems). qRT‐PCR detection of miR‐499a‐5p and U6 (Applied Biosystems) was achieved utilizing TaqMan Universal Master Mix II. After the expression levels were normalized, the relative quantification ($2^{-\Delta \Delta C_{t}}$) was calculated. The sequences of primers and probes were listed in Table S1.

### 2.4 Western blot analysis

RIPA buffer is used to lyse cells on ice to obtain total protein. The SDS‐PAGE gel was used for electrophoresis of an equal amount of various proteins, followed by electrophoresis of the target protein onto the PVDF membrane. Incubate in Tween‐Tris buffered saline (TTBS) embracing 5% skim milk for 2 hours at room temperature then incubate with primary antibody as follows: ZNRD1‐AS1‐144aa‐uORF (1:1000, Beijing Protein Innovation), ELF1 (1:500, Santa Cruz), EMI1 (1:500, Proteintech) and GAPDH (1:5000, Proteintech). After washing with TTBS three times, it was placed in a secondary antibody (goat anti‐rabbit or goat anti‐mouse, 1:4000; Proteintech Group). Immunoblots were subsequently visualized using an ECL (Enhanced Chemiluminescence) kit (Beyotime Institute of Biotechnology). The relative integrated density value (IDV) was calculated using FLuor Chem 2.0 software (Alpha Innotech), which was calculated based on GAPDH as an internal control.

### 2.5 Cell transfection

Short‐hairpin RNAs targeting the ZNRD1‐AS1 (ZNRD1‐AS1(−), sequence: 5′‐TCCTAGGATTGCTGCAGGTCA‐3′), UPF1 (UPF1(−), sequence: 5′‐GATGCAGTTCCGCTCCATT‐3′), UPF2 (UPF2(−), sequence: 5′‐GAAGTTGGTACGGGCACTC‐3′), SMG1 (SMG1(−), sequence: 5′‐GTGTATGTGCGCCAAAGTA‐3′) and EMI1 (EMI1(−), sequence: 5′‐AAAGCCTCAAAGCCTGTAT‐3′) gene and their non‐targeting sequences (negative control, NC) were designed and synthesized, respectively (GeneChem). Short‐hairpin RNA (ELF1(−), sequence: 5′‐GAAAGAGAACACTGAGAAA‐3′) against ELF1 and its non‐targeting sequences (negative control, NC; GenePharama) were designed and synthesized. The 144aa‐uORF full‐length plasmid (144aa‐uORF(+)) and its non‐targeting sequence (negative control, NC; GeneChem) were designed and synthesized. UPF1(−), UPF2(−), SMG1(−) and NC plasmids were stably transfected into 144aa‐uORF plasmid stably transfected glioma cells, respectively. The full‐length ELF1 plasmid (ELF1(+)) and its non‐targeting sequence (negative control, NC; GeneScript) were designed and synthesized. About 24‐well plates (Corning) were supplied for seeding cells that transfected by application of Lipofectamine 3000 reagent (Life Technologies Corporation) in term of the instructions. Screening of stably transfected cells was done by G418 (0.4 mg/mL) or puromycin (1 μg/mL). Underexpression and overexpression efficiencies were examined by qRT‐PCR. To assess the effect of miR‐499a‐5p, agomiR‐499a‐5p (miR‐499a‐5p(+)), antagomiR‐499a‐5p (miR‐499a‐5p(−), sequence: 5′‐AAACAUCACUGCAAGUCUUAA‐3′) and its negative control sequence (miR‐499a‐5p(+)NC and miR‐499a‐5p(−)NC, GenePharma) was transiently transfected into glioma cells. At the same time, they were transiently transfected into glioma cells stably transfected with ZNRD1‐AS1 or ELF1 plasmid. Transiently transfected cells were collected over 48 hours.

### 2.6 Cell proliferation assay

The use of the Counting Cell Kit‐8 (CCK‐8, Dojin) assay was to assess cell proliferation. At a density of 2000 cells per well, U87 and U251 cells were transplanted into 96‐well plates. After 72 hours, 10 μL of CCK‐8 reagent was injected into all wells and cultured at 37°C for 2 hours. The absorbance at 450 nm was examined via a SpectraMax M5 plate reader (Molecular Devices).

### 2.7 Cell migration and invasion assay

Cells (2 × 10^5^) were injected in 100 μL of serum‐free medium to make a cell suspension and inoculated into the upper chamber for migration (or precoated with 80 μL Matrigel solution [BD] for invasion), and 600 μL of medium mixed 10% FBS was injected to the lower chamber. Twenty‐four hours waiting to fix cells those have migrated or invaded the underside of the membrane and stain the cells with 10% Giemsa. Three fields of view were randomly selected under the microscope for counting and photographing.

### 2.8 In vitro VM tube formation assay

About 350 μL of Matrigel solution was placed in each well of a 24‐well plate and placed at 37°C until it solidified. About 500 μL of serum‐free medium containing 3 × 10^5^ cells was injected into all wells and nurtured at 37°C. After 24 hours, photographs were obtained under an inverted microscope (Olympus), and three fields of view were randomly selected to calculate the number of VM tube structures.

### 2.9 CD34‐periodic acid‐schiff (PAS) dual staining

Paraffin‐embedded tissue sections were deparaffinized using xylene, soaked in gradient ethanol and then heated to boiling in an EDTA antigen non‐masking reagent. The cooled tissue specimens were wetted with peroxide, blocked with goat serum and nurtured at 4°C using CD34 primary monoclonal antibody (1:50, Proteintech). After 16 hours, tissue specimens were incubated with the secondary antibody for at 37°C 10 minutes. Staining was performed using a DAB kit (MaiXin Biotech), a periodic acid solution, a Schiff solution and haematoxylin. The VM density was calculated by randomly selecting three regions under the microscope.

### 2.10 Chromatin immunoprecipitation assay

Chromatin immunoprecipitation (ChIP) kit (Beyotime Institute of Biotechnology) was employed in terms of the instructions. The glioma cells were cross‐linked with 1% formaldehyde and uniformly mixed in the lysis solution. Micrococcal nucleases are used to digest chromatin. About 2% of the lysate was taken as an input reference, and the remaining immunoprecipitated samples were incubated with anti‐ELF1 antibody or normal rabbit IgG overnight at 4°C. The elimination of DNA cross‐linking was achieved by 5 mol/L NaCl, as well as proteinase K. Next to purify the DNA. ELF1, PCR1 (F) 5′‐TGTGATAGGTCTGCGATAAGAA‐3′, (R) 5′‐ATAAAGGGCAAGGGGACATT‐3′; PCR2 (F) 5′‐TCCCTAAAGGACAGGACTTTG‐3′, (R) 5′‐CCCGTAAACGCACCTGAG‐3′; PCR3 (F) 5′‐AAACCCAGGGGAGCGTAG‐3′, (R) 5′‐CATCTCCCAGGGCCTTCC‐3′: using the above primers to amplify the DNA.

### 2.11 Reporter vector constructs and luciferase reporter assay

To construct a dual‐luciferase vector (GenePharma), the sequence of potential binding of the ZNRD1‐AS1 gene, the ELF1 gene and their mutant to the miR‐499a‐5p was amplified by PCR, synthesized and cloned into the pmirGlo dual‐luciferase vector (Promega). HEK293T cells were cultured in 96‐well plates with ZNRD1‐AS1‐Wt (or ZNRD1‐AS1‐Mut) or ELF1‐Wt (or ELF1‐Mut) and miR‐499a‐5p(+) or miR‐499a‐5p(+)NC plasmids were cotransfected. After 48 hours, luciferase activity was defined using Dual‐Luciferase reporter assay kit (Promega).

### 2.12 RNA immunoprecipitation

RNA pull‐down assay used Pierce Magnetic RNA‐Protein Pull‐Down Kit (Thermo fisher) in terms of the instructions for use. ZNRD1‐AS1 or antisense RNA was labelled with biotin and co‐incubated with the cell's protein extract and magnetic beads. The resulting bead‐RNA‐protein compound was centrifuged at slow speed and washed with Handee spin column. The sodium dodecyl sulphate buffer was added to the bead compound and boiled. The target protein was measured by Western blot with GAPDH as a control.

### 2.13 Nascent RNA capture

The nascent was detected using a Click‐iT^®^ NascentRNA Capture Kit (Thermo Fisher Scientific) according to the instructions. Briefly, nascent RNA was labelled with 0.2 mmol/L 5‐ethynyl uridine (EU), followed by magnetic beads to capture EU‐neonatal RNA. qRT‐PCR was performed to detect target RNA.

### 2.14 RNA stability assay

For the purpose of inhibiting the synthesis of de novo RNA synthesis, actinomycin D (5 μg/mL) was added to the cell culture medium. Total RNA was obtained at 0, 1, 2, 3, 4 and 5 hours, and its content was assessed through qRT‐PCR. The ratio of the level of RNA at a certain point in time to the level of zero time‐point is used to determine the half‐life.

### 2.15 Xenograft mouse model in vivo

For in vivo studies, 4 weeks old BALB/c (athymic nude mice) were obtained from the Institute of Oncology, Chinese Academy of Medical Sciences. Six randomized groups of nude mice were performed: control group, ZNRD1‐AS1(−)NC+144aa‐uORF(+)NC+ELF1(−)NC, ZNRD1‐AS1(−), 144aa‐uORF(+), ELF1(−) and ZNRD1‐AS1(−)+144aa‐uORF(+)+ELF1(−) group. All in vivo studies are entirely in conformity with the animal experiment procedures adopted by the Ethics Committee of the Chinese Medical University. Glioma cells (3 × 10^5^) were injected into the subcutaneous region of the right abdomen of nude mice. At 7 days intervals, tumour volume was measured in terms of the estimation formula: volume (mm^3^) = length × width^2^/2. The density of the VM of the nude mouse model was measured by CD34‐PAS dual staining. Survival analysis was obtained by transplanting glioma cells (3 × 10^5^) into the right striatum of nude mice. Survival analysis was performed using Kaplan‐Meier survival curves.

### 2.16 Statistical analysis

All data were expressed as mean ± SD and from at least three independent experiments. Statistical analysis of Student's *t* test or one‐way ANOVA was executed by GraphPad Prism v5.01 (GraphPad Software) software. When *P* < .05, the difference was deemed to be statistically significant.

## 3 RESULT

### 3.1 ZNRD1‐AS1 is highly expressed in glioma tissues and cells, and down‐regulation of ZNRD1‐AS1 inhibits VM formation in glioma cells

**FIGURE 1:**
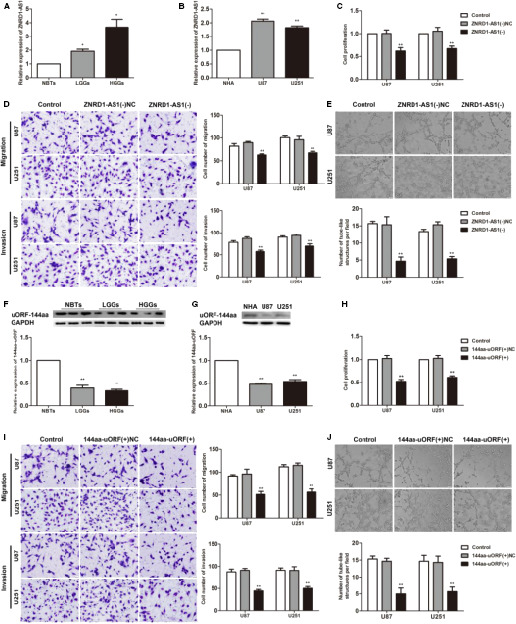
The expression and effect of ZNRD1‐AS1 and 144aa‐uORF on the biological behaviour of glioma cells. A, ZNRD1‐AS1 expression levels in non‐tumorous brain tissues (NBTs), low‐grade glioma tissues (WHO I‐II) and high‐grade glioma tissues (WHO III‐IV). Data are presented as the mean ± SD (n = 12 in each group). ^*^ *P* < .05, vs NBTs group. B, ZNRD1‐AS1 expression levels in normal human astrocytes (NHA), U87 and U251 cells. Data are presented as the mean ± SD (n = 3 in each group). ^**^ *P* < .01 vs NHA group. C, H, CCK‐8 assay was conducted to investigate the effect of ZNRD1‐AS1 (144aa‐uORF) on proliferation of U87 and U251 cells. D, I, Transwell assays were used to measure the effect of ZNRD1‐AS1 (144aa‐uORF) on cell migration and invasion of U87 and U251 cells. Scale bars represent 20 μm. E, J, Three‐dimensional culture was used to measure the effect of ZNRD1‐AS1 (144aa‐uORF) on cell VM of U87 and U251 cells. Scale bars indicate 50 μm. Data are presented as mean ± SD (n = 3, each group). ^**^ *P* < .01 vs ZNRD1‐AS1(−)NC (144aa‐uORF(+)NC). F, The expression of 144aa‐uORF in glioma tissues of different grades and NBTs. Data are presented as the mean ± SD (n = 12 in each group). ^**^ *P* < .01 vs NBTs group. G, The expression of 144aa‐uORF in NHA, U87 and U251 cells. Data are presented as the mean ± SD (n = 3 in each group); ^**^ *P* < .01 vs NHA group

The expression level of ZNRD1‐AS1 was detected by qRT‐PCR in normal brain tissues (NBTs), low‐grade glioma tissues (Grade I‐II), high‐grade glioma tissues (Grade III‐IV) and NHAs, U87, U251 cell lines. The expression of ZNRD1‐AS1 was significantly elevated in U87 and U251 cells compared with the NHA group (Figure 1A). Compared with NBT, ZNRD1‐AS1 was highly expressed in glioma tissues (Figure 1B). In this study, U87 and U251 cells with knocking down of ZNRD1‐AS1 expression were further constructed to detect the effects of ZNRD1‐AS1 on the proliferation, migration, invasion and VM formation of glioma cells. In Figure 1C, the CCK8 experimental results showed that the proliferation of cells in the ZNRD1‐AS1(−) group was weaker than that in the ZNRD1‐AS1(−)NC group. Transwell results revealed a reduction in the number of migrations and invasions in the ZNRD1‐AS1(−) group compared with the ZNRD1‐AS1(−)NC group (Figure 1D). In vitro VM tube formation experiments showed that the VM formation ability of U87 and U251 cells in the ZNRD1‐AS1(−) group decreased remarkedly compared with the NC(−) group (Figure 1E).

### 3.2 144aa‐uORF is down‐regulated in glioma, promoting the degradation of ZNRD1‐AS1 through the NMD pathway and inhibiting VM formation of glioma cells

**FIGURE 2:**
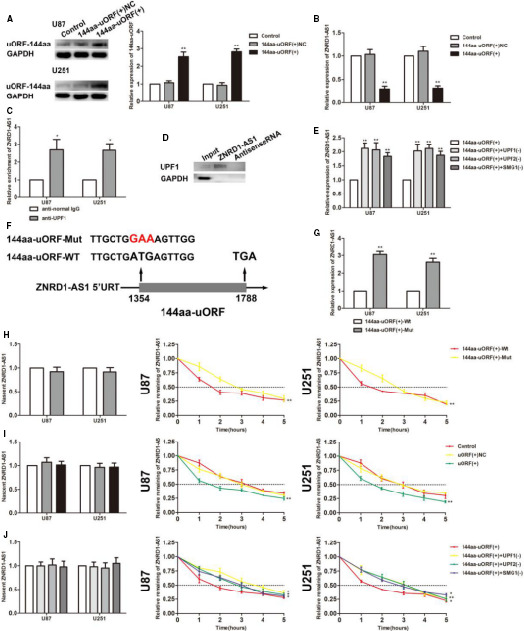
144aa‐uORF suppressed the malignant behaviours of glioma cells by unstabilizing ZNRD1‐AS1 through NMD pathway. A, Western blot was used to investigate the transfection efficiency of 144aa‐uORF. Data are presented as the mean ± SD (n = 3 in each group). ^**^ *P* < .01 vs 144aa‐uORF(+)NC group. B, Real‐time qPCR analysis for 144aa‐uORF regulating ZNRD1‐AS1 expression in U87 and U251 cells. Data are presented as the mean ± SD (n = 3 in each group). ^**^ *P* < .01 vs 144aa‐uORF(+)NC group. C, RNA‐IP confirmed the binding interaction between UPF1 and ZNRD1‐AS1. Relative enrichment was measured by qRT‐PCR. Data are presented as mean ± SD (n = 3, each group). ^**^ *P* < .01 vs antinormal IgG respective group. D, UPF1 and GAPDH protein levels in immunoprecipitation with ZNRD1‐AS1 RNA were evaluated by Western blots. The expression levels of UPF1 and GAPDH proteins are shown. E, Real‐time qPCR analysis for UPF1, UPF2 and SMG1 regulating ZNRD1‐AS1 expression in U87 and U251 cells. Data are presented as the mean ± SD (n = 3 in each group). ^**^ *P* < .01 vs 144aa‐uORF(+) group. F, The initiation codon sequence of 144aa‐uORF and its mutation. G, Real‐time qPCR analysis for 144aa‐uORF(+)‐Mut regulating ZNRD1‐AS1 expression in U87 and U251 cells. Data are presented as the mean ± SD (n = 3 in each group). ^**^ *P* < .01 vs 144aa‐uORF(+)‐Wt group. H, Stability of ZNRD1‐AS1 by 144aa‐uORF(+)‐Mut. Data are presented as mean ± SD (n = 3, each group). ^**^ *P* < .01 vs 144aa‐uORF(+)‐Wt group. I, Stability of ZNRD1‐AS1 by 144aa‐uORF. Data are presented as mean ± SD (n = 3, each group). ^**^ *P* < .01 vs 144aa‐uORF(+)NC group. J, Stability of ZNRD1‐AS1 by UPF1, UPF2 and SMG1. Data are presented as mean ± SD (n = 3, each group). ^*^ *P* < .05, ^**^ *P* < .01 vs 144aa‐uORF(+) group

Compared with NBTs and NHA, 144aa‐uORF was down‐regulated in glioma tissues and cells by Western blot (Figure 1F,G). The U87 and U251 cell lines stably transfected with 144aa‐uORF were further constructed to detect its function on the biological behaviour of glioma cells. The data described that the proliferation, migration, invasion and VM formation in the 144aa‐uORF(+) group were significantly inhibited compared with the 144aa‐uORF(+)NC group (Figure 1H‐J). 144aa‐uORF(+) increased the expression of 144aa‐uORF protein (Figure 2A), while ZNRD1‐AS1 expression was inhibited (Figure 2B).

RNA‐IP experiments were used to verify whether UPF1 bound to ZNRD1‐AS1. As shown in Figure 2C, ZNRD1‐AS1 was dramatically more enriched in the anti‐UPF1 group than in the anti‐IgG group. The results of RNA pull‐down experiments showed that the level of UPF1 detected in the captured portion of ZNRD1‐AS1 was much richer than that of the Antisense RNA group, indicating a binding between UPF1 and ZNRD1‐AS1 (Figure 2D). To determine whether the 144aa‐uORF reduced the stability of ZNRD1‐AS1 via the NMD pathway, UPF1, UPF2 and SMG1 knocked down in U87 and U251 cell lines, respectively, cotransfected with 14aa‐uORF overexpression. The experimental results showed that in the 144aa‐uORF(+)+UPF1(−), 144aa‐uORF(+)+UPF2(−) and 144aa‐uORF(+)+SMG1(−) cells, the expression of ZNRD1‐AS1 was up‐regulated (Figure 2E). Based on the overexpression of 144aa‐uORF, its start codon was mutated. As shown, the expression of ZNRD1‐AS1 in 144aa‐uORF(+)‐Mut group was abundant compared with 144aa‐uORF(+)‐Wt (Figure 2F,G). Compared with 144aa‐uORF(+)‐Wt, 144aa‐uORF(+)‐Mut group had no statistical difference in qRT‐PCR detection of neonatal ZNRD1‐AS1, and the half‐life of ZNRD1‐AS1 was shortened (Figure 2H). 144aa‐uORF(+) group compared with 144aa‐uORF(+)NC group, there was no statistical difference of novel ZNRD1‐AS1 by qRT‐PCR. Same result also found in the 144aa‐uORF(+)+UPF1(−), 144aa‐uORF(+)+UPF2(−) and 144aa‐uORF(+)+SMG1(−) comparing with the 144aa‐uORF(+) group. The half‐life of ZNRD1‐AS1 in the 144aa‐uORF(+) group was shortened compared with the 144aa‐uORF(+)NC group. The 144aa‐uORF(+)+UPF1(−), 144aa‐uORF(+)+UPF2(−) and 144aa‐uORF(+)+SMG1(−) groups extended the half‐life of ZNRD1‐AS1 compared with the 144aa‐uORF(+) group (Figure 2I,J).

### 3.3 miR‐499a‐5p is reduced in glioma tissues and cells, and ZNRD1‐AS1 binds to miR‐499a‐5p to regulate VM formation

**FIGURE 3:**
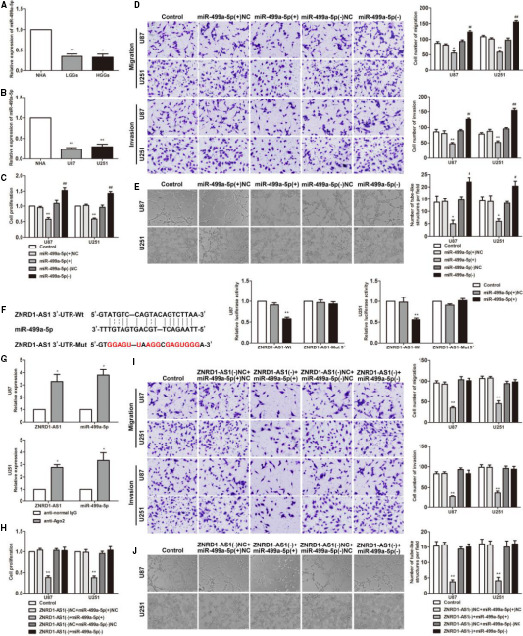
The expression and effect of miR‐499a‐5p on the biological behaviour of glioma cells; miR‐499a‐5p mediated the tumour‐suppressive effects of ZNRD1‐AS1 knockdown on glioma cell lines. A, The expression levels of miR‐499a‐5p in glioma tissues. Data are presented as the mean ± SD (n = 12 in each group). ^*^ *P* < .05, ^**^ *P* < .01 vs NBTs group; B, miR‐499a‐5p expression levels in glioma cells. Data are presented as the mean ± SD (n = 3 in each group). ^**^ *P* < .01 vs NHA group. C, CCK‐8 assay was conducted to investigate the effect of miR‐499a‐5p on proliferation of U87 and U251 cells. D, Transwell assays were used to measure the effect of miR‐499a‐5p on cell migration and invasion of U87 and U251 cells. Scale bars represent 20 μm. E, Three‐dimensional culture was used to measure the effect of miR‐499a‐5p on cell VM of U87 and U251 cells. Scale bars indicate 50 μm. Data are presented as mean ± SD (n = 3, each group). ^*^ *P* < .05, ^**^ *P* < .01 vs miR‐499a‐5p(+)NC; ^#^ *P* < .05, ^##^ *P* < .01 vs miR‐499a‐5p(−)NC. F, Relative luciferase activity was performed by dual‐luciferase reporter assay. Error bars represent as the mean ± SD (n = 3, each group). ^**^ *P* < .01 vs miR‐499a‐5p(+)NC. G, miR‐499a‐5p was identified in the ZNRD1‐AS1‐RISC complex. ZNRD1‐AS1 and miR‐499a‐5p enrichment were measured using qRT‐PCR. Error bars represent as the mean ± SD (n = 3, each group). ^*^ *P* < .05, ^**^ *P* < .01 vs anti‐IgG group. H, CCK‐8. I, Transwell. Scale bars represent 20 μm. J, Three‐dimensional culture. Data are presented as the mean ± SD (n = 3 in each group). Scale bars represent 50 μm. ^**^ *P* < .01 vs ZNRD1‐AS1(−)NC+miR‐499a‐5p(+)NC group

The results of miRNA microarray analysis confirmed that miR‐499a‐5p was significantly up‐regulated in glioma cells with ZNRD1‐AS1 knockdown, indicating that miR‐499a‐5p may be involved in the regulation of glioma cells induced by ZNRD1‐AS1 (Figure S1). The statistics confirmed that the expression of miR‐499a‐5p in glioma tissues and cells was higher than in NBTs and NHA (Figure 3A,B). U87 and U251 cell lines were treated with miR‐499a‐5p(+) and miR‐499a‐5p(−), respectively, to examine the impacts on the biological behaviour of glioma cells. Our statistics confirmed that the miR‐499a‐5p(+) group had lower proliferation, migration, invasion and VM formation ability than the miR‐499a‐5p(+)NC group. The miR‐499a‐5p(−) group had higher proliferation, migration, invasion and VM formation ability than the miR‐499a‐5p(−)NC group (Figure 3C‐E).

With the help of bioinformatics database (Starbase), it is predicted that ZNRD1‐AS1 and miR‐499a‐5p have possible binding sites. The binding of the two was verified by dual‐luciferase reporter assay. There was a decrease in the activity of luciferase in the ZNRD1‐AS1‐Wt+miR‐499a‐5p(+) group compared with that in the ZNRD1‐AS1‐Wt+miR‐499a‐5p(+)NC group. The luciferase activity of the ZNRD1‐AS1‐Mut5′+miR‐499a‐5p(+) group was not statistically different from that of the ZNRD1‐AS1‐Mut5′+miR‐499a‐5p(+)NC group (Figure 3F). As RNA‐IP assay expressed, in the anti‐Ago2 group, the enrichment of ZNRD1‐AS1 and miR‐499a‐5p was especially enhanced compared with the anti‐IgG group, indicating ZNRD1‐AS1 and miR‐499a‐5p were RNA‐induced silencing complexes (RISC; Figure 3G), respectively. The cell lines stably transfected with ZNRD1‐AS1(−) was transfected with miR‐499a‐5p(+) and miR‐499a‐5p(−) to detect the biological behaviour of glioma cells. As the figures illustrate, the ZNRD1‐AS1(−)+miR‐499a‐5p(+) group reduced proliferation, migration, invasion and VM formation ability compared with the ZNRD1‐AS1(−)NC+miR‐499a‐5p(+)NC group. For ZNRD1‐AS1(−)NC+miR‐499a‐5p(+)NC, ZNRD1‐AS1(−)NC+miR‐499a‐5p(−)NC and ZNRD1‐AS1(−)+miR‐499a‐5p(−), comparison of the three groups with the control group revealed no statistical difference (Figure 3H‐J).

### 3.4 ELF1 is up‐regulated in glioma tissues and cells, and miR‐499a‐5p binds to ELF1 to regulate VM formation

**FIGURE 4:**
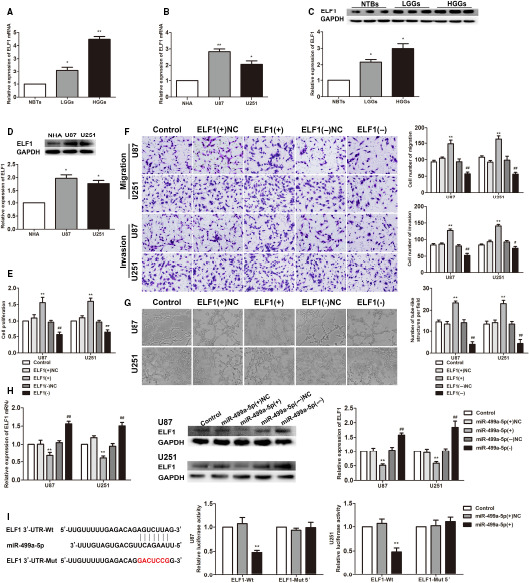
ELF1 was up‐regulated in glioma tissues and cells and exerted oncogenic function in glioma cells. A, C, ELF1 mRNA and protein expression levels in NBTs and glioma tissues. Data are presented as the mean ± SD (n = 12 in each group). ^*^ *P* < .05, ^**^ *P* < .01 vs NBTs group. B, D, ELF1 mRNA and protein expression levels in NHA and glioma cells. Data are presented as the mean ± SD (n = 3 in each group). ^*^ *P* < .05, ^**^ *P* < .01 vs NHA group. E, CCK‐8 assay was used to explore the effect of ELF1 on proliferation in U87 and U251 cells. F, Transwell assays were used to measure the effect of ELF1 on cell migration and invasion of U87 and U251 cells. Scale bars indicate 20 μm. Data are presented as mean ± SD (n = 3, each group). ^**^ *P* < .01 vs ELF1(+)NC; ^#^ *P* < .05, ^##^ *P* < .01 vs ELF1(−)NC. G, Three‐dimensional culture was used to measure the effect of ELF1 on cell VM of U87 and U251 cells. Scale bars indicate 50 μm. H, qRT‐PCR and Western blot assay were used to detect the ELF1 expression after miR‐499a‐5p overexpression or knockdown. ^**^ *P* < .01 vs miR‐499a‐5p(+)NC group; ^##^ *P* < .01 vs miR‐499a‐5p(+)NC group. I, Relative luciferase activity was performed by dual‐luciferase reporter assay. Error bars represent as the mean ± SD (n = 3, each group). ^**^ *P* < .01 vs miR‐499a‐5p(+)NC group

In glioma tissues and cells, the ELF1 expression was detected by qRT‐PCR and Western blot. As shown in Figure 4A‐D, the expression levels of ELF1 mRNA and protein were obviously increased, compared with normal tissues and cells. The cell lines were treated, respectively, with ELF1(+) and ELF1(−), to examine their effects on cell biological behaviour. The results showed that ELF1(+) enhanced the proliferation, migration, invasion and VM abilities in cells compared with the ELF1(+)NC group, whereas the opposite result was expressed in ELF1(−) group (Figure 4E‐G). miR‐499a‐5p(+) reduced the ELF1 expression, while miR‐499a‐5p(−) increased that (Figure 4H).

Through the analysis of bioinformatics databases (TargetScan), there existed a potential binding site for ELF13′UTR and miR‐499a‐5p. The results of dual‐luciferase reporter assay clarified that there was a decrease in the activity of luciferase in the ELF1‐Wt+miR‐499a‐5p(+) group compared with that in the ELF1‐Wt+miR‐499a‐5p(+)NC group, while the change in ELF‐Mut5′+miR‐499a‐5p(+) group was not statistically significant (Figure 4I).

### 3.5 ZNRD1‐AS1 competitively binds to miR‐499a‐5p and negatively regulates the inhibitory effect of miR‐499a‐5p on ELF1

**FIGURE 5:**
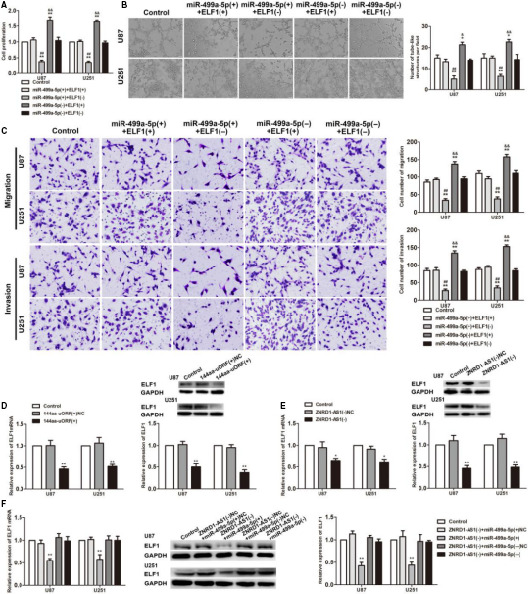
ELF1 was involved in miR‐499a‐5p mediated tumour‐suppressive function in glioma cells. A, CCK‐8 assays were performed on U87 and U251 cells with the altered expression of miR‐499a‐5p and ELF1. B, Three‐dimensional culture was used to on U87 and U251 cells with the altered expression of miR‐499a‐5p and ELF1. Scale bars indicate 50 μm. C, Quantification number of migration and invasion cells with the altered expression of miR‐499a‐5p and ELF1. Scale bars represent 20 μm. Data are presented as the mean ± SD (n = 3 in each group). ^*^ *P* < .05, ^**^ *P* < .01 vs control group; ^##^ *P* < .01 vs miR‐499a‐5p(+)+ELF1(+) group. ^&^ *P* < .05, ^&&^ *P* < .01 vs miR‐499a‐5p(−)+ELF1(−) group. D, qRT‐PCR assay and Western blot analysis were used to detect the ELF1 expression after 144aa‐uORF(+) overexpression. Data are presented as the mean ± SD (n = 3 in each group). ^*^ *P* < .05, ^**^ *P* < .01 vs 144aa‐uORF(+)NC group. E, qRT‐PCR assay and Western blot analysis were used to detect the ELF1 expression after ZNRD1‐AS1 knockdown. Data are presented as the mean ± SD (n = 3 in each group). ^*^ *P* < .05, ^**^ *P* < .01 vs ZNRD1‐AS1(−)NC group. F, mRNA and protein levels of ELF1 regulated by ZNRD1‐AS1 and miR‐499a‐5p in U87 and U251 cells. Data are presented as the mean ± SD (n = 3 in each group). ^**^ *P* < .01 vs ZNRD1‐AS1(−)NC+miR‐499a‐5p(+)NC group

To verify whether ELF1 participates in the management of glioma cells VM via the ZNRD1‐AS1/miR‐499a‐5p pathway, and to detect the effect of ELF1 on the biological behaviour of glioma cells, cotransfection of miR‐499a‐5p cell lines with ELF1 was constructed. miR‐499a‐5p(+)+ELF1(−) group was compared with control group and miR‐499a‐5p(+)+ELF1(+) group, glioma cell proliferation, VM formation, migration and invasion of glioma cells were clearly decreased. Compared with the control group and the miR‐499a‐5p(−)+ELF1(−) group, the proliferation, VM formation, migration and invasion ability of the miR‐499a‐5p(−)+ELF1(+) group were significantly enhanced. The miR‐499a‐5p(+)+ELF1(+) group and the miR‐499a‐5p(−)+ELF1(−) group had no different from the control group (Figure 5A‐C).

Changes in ELF1 mRNA and protein expression were detected in 144aa‐uORF(+), ZNRD1‐AS1(−) and cotransfection of ZNRD1‐AS1 and miR‐499a‐5p glioma cell lines. Compared with the 144aa‐uORF(+) (ZNRD1‐AS1(−)NC) group, the 144aa‐uORF(+) (ZNRD1‐AS1(−)) group decreased the mRNA and protein expression of ELF1 (Figure 5D,E). The expression of ELF1 was lower in the ZNRD1‐AS1(−)+miR‐499a‐5p(+) group than in the ZNRD1‐AS1(−)NC+miR‐499a‐5p(+)NC group. For ZNRD1‐AS1(−)NC+miR‐499a‐5p(+)NC, ZNRD1‐AS1(−)NC+miR‐499a‐5p(−)NC and ZNRD1‐AS1(−)+miR‐499a‐5p(−), the statistical difference between the three groups was not obviously with the comparison of the control group (Figure 5F).

### 3.6 The expression of EMI1 is increased in glioma tissues and cells, and ELF1 directly binds to the promoter of EMI1 to regulate VM formation

**FIGURE 6:**
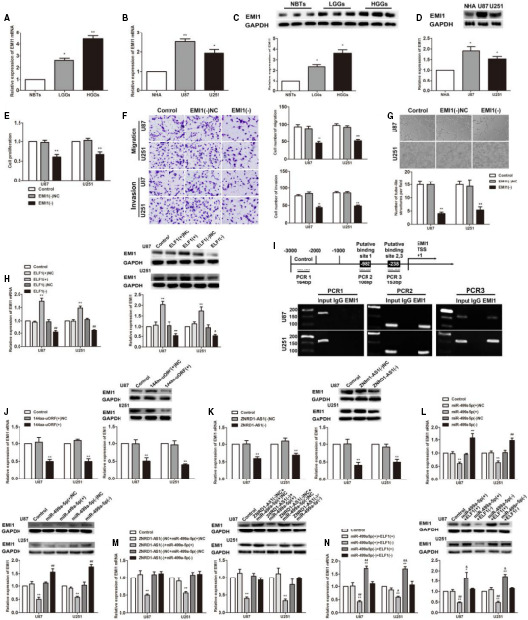
EMI1 was up‐regulated in glioma tissues and cells and played oncogenic role in glioma cells. A, C, EMI mRNA and protein expression levels in NBTs and glioma tissues. Data are presented as the mean ± SD (n = 12 in each group). ^*^ *P* < .05, ^**^ *P* < .01 vs NBTs group. B, D, EMI1 mRNA and protein expression levels in NHA, U87 and U251 cells. Data are presented as the mean ± SD (n = 3 in each group). ^*^ *P* < .05, ^**^ *P* < .01 vs NHA group. E, CCK‐8. F, Transwell. Scale bars represent 20 μm. G, Three‐dimensional culture. Data are presented as the mean ± SD (n = 3 in each group). Scale bars represent 50 μm. ^*^ *P* < .05, ^**^ *P* < .01 vs EMI1(−)NC group. H, qRT‐PCR and Western blot analysis for ELF1 regulating EMI1 expression in U87 and U251 cells. Data are presented as the mean ± SD (n = 3 in each group). ^**^ *P* < .01 vs ELF1(+)NC group; ^#^ *P* < .05, ^##^ *P* < .01 vs ELF1(−)NC group. I, ELF1 bound to the promoter of EMI1 in U87 and U251 glioma cells. Putative ELF1 binding sites are indicated. Immunoprecipitated DNA was amplified by PCR. Normal rabbit IgG was used as a negative control. J, Real‐time qPCR analysis and Western blot assay were used to detect the EMI1 expression after 144aa‐uORF(+) overexpression. Data are presented as the mean ± SD (n = 3 in each group). ^*^ *P* < .05, ^**^ *P* < .01 vs 144aa‐uORF(+)NC group. K, Real‐time qPCR analysis and Western blot assay were used to detect the EMI1 expression after ZNRD1‐AS1 knockdown. Data are presented as the mean ± SD (n = 3 in each group). ^*^ *P* < .05, ^**^ *P* < .01 vs ZNRD1‐AS1(−)NC group. L, Real‐time qPCR analysis and Western blot assay were used to detect the EMI1 expression after miR‐499a‐5p overexpression or knockdown. Data are presented as the mean ± SD (n = 3 in each group). ^*^ *P* < .05, ^**^ *P* < .01 vs miR‐499a‐5p(−)NC group; ^##^ *P* < .01 vs miR‐499a‐5p(+)NC group. M, mRNA and protein levels of EMI1 regulated by ZNRD1‐AS1 and miR‐499a‐5p in U87 and U251 cells. Data are presented as the mean ± SD (n = 3 in each group). ^**^ *P* < .01 vs ZNRD1‐AS1(−)NC+miR‐499a‐5p(+)NC group. N, mRNA and protein levels of EMI1 regulated by miR‐499a‐5p and ELF1 in U87 and U251 cells. Data are presented as the mean ± SD (n = 3 in each group). ^*^ *P* < .05, ^**^ *P* < .01 vs control group; ^#^ *P* < .01, ^##^ *P* < .01 vs miR‐499a‐5p(+)+ELF1(+) group. ^&^ *P* < .05, ^&&^ *P* < .01 vs miR‐499a‐5p(−)+ELF1(−) group

According to qRT‐PCR and Western blot results, EMI1 was overexpressed in glioma tissues and cells (Figure 6A‐D). Detection of EMI1(−) treated cell lines revealed a significant decrease in glioma cell proliferation, migration, invasion and VM formation ability (Figure 6E‐G). ELF1(+) increased expression of EMI1 compared with the ELF1(+)NC group. However, ELF1(−) group has the opposite phenomenon (Figure 6H). Using bioinformatic databases (JASPAR) analysis, it was showed that there was a potential binding site for binding to ELF1 in the promoter region of EMI1. The results of the ChIP experiment demonstrated the binding of ELF1 and EMI1 (Figure 6I).

We further constructed 144aa‐uORF(+), ZNRD1‐AS1(−), miR‐499a‐5p up‐regulation and knockdown, ZNRD1‐AS1 and miR‐499a‐5p cotransfection, and miR‐499a‐5p and ELF1 cotransfection into glioma cells. The change in EMI1 expression was measured. In comparison with the 144aa‐uORF(+)NC (ZNRD1‐AS1(−)NC) group, expression of EMI1 exhibited a decrease in the 144aa‐uORF(+) (ZNRD1‐AS1(−)) group (Figure 6J,K). miR‐499a‐5p(+) inhibited EMI1 expression, compared with miR‐499a‐5p(+)NC, whereas miR‐499a‐5p(−) promote EMI1 expression, compared with miR‐499a‐5p(−)NC (Figure 6L). The mRNA and protein expression of EMI1 was decreased in the ZNRD1‐AS1(−)+miR‐499a‐5p(+) group compared with the ZNRD1‐AS1(−)NC+miR‐499a‐5p(+)NC group. For ZNRD1‐AS1(−)NC+miR‐499a‐5p(+)NC, ZNRD1‐AS1(−)NC+miR‐499a‐5p(−)NC and ZNRD1‐AS1(−)+miR‐499a‐5p(−), the statistical difference between the three groups was not obviously with the comparison of the control group (Figure 6M). Compared with the control group and the miR‐499a‐5p(+)+ELF1(+) group, the miR‐499a‐5p(+)+ELF1(−) group achieved a decrease in the expression of EMI1, whereas the miR‐499a‐5p(−)+ELF1(+) group gained an increase compared with the control group and the miR‐499a‐5p(−)+ELF1(−) group, respectively. No significant difference was found between the miR‐499a‐5p(+)+ELF1(+) group and the miR‐499a‐5p(−)+ELF1(−) group compared with the control group (Figure 6N).

### 3.7 The separate and combined application of ZNRD1‐AS1(−), 144aa‐uORF(+) and EMI1(−) restricted tumour growth in nude mice and prolonged survival in nude mice

**FIGURE 7:**
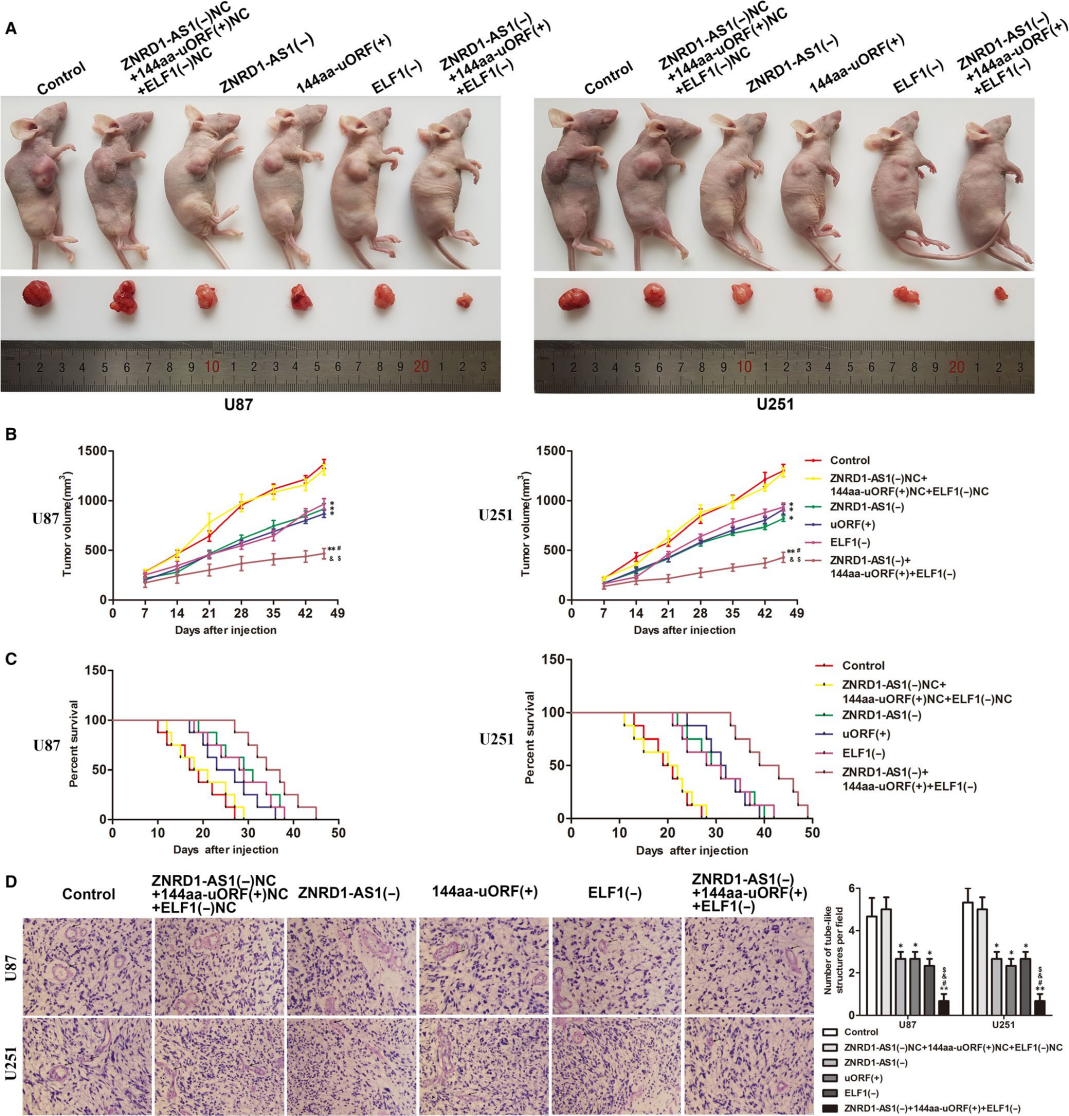
The stable expressing cells were used for tumour xenografts study in vivo. A, The nude mice sample tumour from respective group was shown. B, Tumour growth curves in nude mice were shown. Tumour volume was calculated every 7 d after injection, and tumour was excised after 46 d; data are presented as mean ± SD (n = 8, each group). ^*^ *P* < .05, ^**^ *P* < .01 vs ZNRD1‐AS1(−)NC+144aa‐uORF(+)NC+ELF1(+)NC group, ^#^ *P* < .05 vs ZNRD1‐AS1(−) group, ^&^ *P* < .05 vs 144aa‐uORF(+) group, ^&^ *P* < .05 vs ELF1(−) group. C, The survival curves of nude mice that were injected into the right striatum were shown (n = 8, each group). D, CD34‐PAS staining was used to detect the VM in xenografted tumour. Data are presented as mean ± SD (n = 3, each group). Scale bars indicate 20 μm. ^*^ *P* < .05, ^**^ *P* < .01 vs ZNRD1‐AS1(−)NC+144aa‐uORF(+)NC+ELF1(+)NC group, ^#^ *P* < .05 vs ZNRD1‐AS1(−) group, ^&^ *P* < .05 vs 144aa‐uORF(+) group, ^&^ *P* < .05 vs ELF1(−) group

Compared with the control group, subcutaneous xenograft experiments in nude mice indicated that the tumour volume of the ZNRD1‐AS1(−) group, the 144aa‐uORF(+) group and the EMI1(−) group was significantly limited, and the survival time became longer. Compared with ZNRD1‐AS1(−), 144aa‐uORF(+) and EMI1(−) alone, the combination of the three produced minimal tumour volume (Figure 7A,B). The results of intracranial striatum orthotopic transplantation revealed that the survival time of ZNRD1‐AS1(−) group, 144aa‐uORF(+) group and EMI1(−) group was longer than that of control group. The nude mice that were used in combination obtained the longest survival time (Figure 7C). CD34‐periodic acid‐schiff (PAS) dual staining clearly revealed the density of VM in ZNRD1‐AS1(−) group, 144aa‐uORF(+) group and EMI1(−) group was lower than ZNRD1‐AS1(−)NC, 144aa‐uORF(+)NC and EMI1(−)NC cotransfection group, the combined group of ZNRD1‐AS1(−), 144aa‐uORF(+) and EMI1(−) obtained the smallest VM density(Figure 7D).

## 4 DISCUSSION

Our study indicated that ZNRD1‐AS1‐144aa‐uORF and miR‐499a‐5p were underexpressed in glioma tissues and cells, and ZNRD1‐AS1, ELF1 and EMI1 were highly expressed. Overexpression of 144aa‐uORF reduced the stability of ZNRD1‐AS1 and inhibited its expression through the NMD pathway, thereby interfering with cell proliferation, migration, invasion and VM formation ability. Down‐regulation of ZNRD1‐AS1 enhanced the negative regulation of miR‐499a‐5p on ELF1 and decreased the expression of ELF1 through targeting combined with miR‐499a‐5p. Furthermore, this down‐regulation reduces the transcriptional regulation of ELF1 on EMI1 and inhibits cell proliferation, migration, invasion and VM. The results of this essay demonstrated for the first time the ability of 144aa‐uORF to regulate the formation of glioma cell VM via the ZNRD1‐AS1/miR‐499a‐5p/ELF1/EMI1 pathway.

Upstream open reading frame is widely present in the 5′UTR of eukaryotic RNA and is a cis‐regulatory sequence element prevalent in transcriptional leader sequences.^25^ Our results showed that ZNRD1‐AS1‐144aa‐uORF was minimally expressed in glioma tissues, U87 and U251, and further showed that up‐regulation of 144aa‐uORF inhibited glioma cell proliferation, migration, invasion and VM. The data suggest 144aa‐uORF played a role as a tumour suppressor gene. Similar to our results, the low expression of NR2C2‐47aa‐uORF in gliomas inhibited cell malignant biological behaviour, but promoted apoptosis.^26^ LncRNAs are a sort of non‐coding RNAs with multiple regulatory functions. Earlier studies have shown that lncRNAs participate in regulating the occurrence, development and VM formation of various tumours, including gastric cancer, hepatocellular carcinoma and gliomas.^27^, ^28^, ^29^ This study found that expression of ZNRD1‐AS1 was elevated in glioma tissues and cells, and knockdown of ZNRD1‐AS1 inhibited glioma cell VM. As was reported earlier, SNHG16 and linc00667 are increased in gliomas, which enhances cell VM formation ability.^30^ LncRNA HOXA‐AS2 is up‐regulated in gliomas and promotes VM formation.^31^

Upstream open reading frames typically reduce the expression of RNA proteins by inhibiting the translation of the main ORFs in their associated RNA.^32^ Previous studies have indicated that uORFs could alter the stability of RNA. For example, uORFs can cause a reduction in half‐life and reduce the stability of RNA.^33^ According to our further studies, up‐regulation of 144aa‐uORF shortened the half‐life of ZNRD1‐AS1, decreased the stability of ZNRD1‐AS1 and inhibited its expression. After overexpression of 144aa‐uORF, a mutation in the initiation codon ATG (GAA) was performed such that the 144aa‐uORF could not be translated. Then, the inhibition of the stability and expression of ZNRD1‐AS1 by 144aa‐uORF alone was reversed. Related pieces of literature have reported that longer (50aa and longer) uORFs in plants activate NMD to reduce RNA stability.^34^ Human genomic analysis indicates that short open reading frame (ORF), presenting in most lncRNAs, may activate the NMD pathway to degrade lncRNA.^35^ The 5′UTR uORF can be regarded as a premature termination codon (PTC),^36^, ^37^ which promotes the degradation of lncRNA by activating NMD when the uORF is translated.^38^ The NMD pathway acts as a regulatory mechanism for RNA by promoting the degradation of PTC‐containing RNA.^39^ The process of uORF activating NMD can be summarized as follows: when uORF is translated, its termination codon is recognized as a PTC by stagnate ribosomes, while factors such as UPF1 and SMG1 are recruited to stagnant ribosomes to form the SURF complex (SMG1–UPF1–eRF1–eRF3). Subsequently, UPF1 was activated by UPF2 and other factors to promote the degradation of RNA.^40^ In this study, RIP and RNA pull‐down showed that UPF1 binds to ZNRD1‐AS1, indicating that ZNRD1‐AS1 was the target of the NMD pathway. When 144aa‐uORF is up‐regulated, the respective inhibition of UPF1, UPF2 and SMG1 (key factors in the NMD pathway) reverses the inhibitory effect of 144aa‐uORF up‐regulation on ZNRD1‐AS1. These results showed that the mechanism underlying the inhibition of the stability and expression of ZNRD1‐AS1 by the 144aa‐uORF was dependent on the NMD pathway. The above results suggested that ZNRD1‐AS1‐144aa‐uORF could reduce the stability of ZNRD1‐AS1 and its expression by activating the NMD pathway, thereby inhibiting the formation of cell VM. Related studies have clarified that the uORF of CDKN1A mRNA could activate the NMD pathway, reduce the stability of CDKN1A and inhibit its expression.^8^

Our current data clarified that miR‐499a‐5p was decreased, and overexpression of miR‐499a‐5p could negatively regulate glioma cell proliferation, migration, invasion and VM. The knockdown of miR‐499a‐5p had the opposite effect. Some similar studies have found that miR‐499a‐5p acts as a tumour suppressor in a variety of cancers. In oesophageal cancer, miR‐499a‐5p inhibits cell proliferation and promotes apoptosis.^41^ miR‐499a‐5p is underexpressed in non‐small cell lung cancer, and overexpression of miR‐499a‐5p inhibits cell proliferation and promotes apoptosis.^42^ In hepatocellular carcinomas, overexpression of miR‐499a‐5p inhibits cell migration and invasion.^43^ In our study, ZNRD1‐AS1 and miR‐499a‐5p had opposite effects on the biological behaviour of glioma cells. The potential binding sites between miR‐499a‐5p and ZNRD1‐AS1 were predicted by biological information analysis, and the binding effect was proved by a dual‐luciferase reporter gene. We further proposed that ZNRD1‐AS1 bound and negatively regulated miR‐499a‐5p expression in a RISC‐dependent manner. Knockdown of miR‐499a‐5p reversed the inhibitory effect of ZNRD1‐AS1 knockdown on VM formation in glioma cells. ZNRD1‐AS1 promoted glioma cell VM by targeting miR‐499a‐5p and inhibiting its expression. Another study reports that SNHG5 binds to miR‐32 in gastric cancer and inhibits the expression of miR‐32 in a RISC‐dependent manner, enhancing the ability of cell proliferation and migration.^44^

In the current experiment, it was elucidated that ELF1 was largely abundant in gliomas. Up‐regulation of ELF1 enhanced the VM ability of glioma cell, whereas knockdown of ELF1 resulted in the opposite. The data argued that ELF1 might have a function in promoting oncogenic genes in gliomas. Interestingly, ELF1 is increased in non‐small cell lung cancer (NSCLC) and further cell metastasis, invasion and VM.^45^ The binding site between miR‐499a‐5p and ELF1 3′UTR was found by a dual‐luciferase reporter gene. Up‐regulation of miR‐499a‐5p decreased the expression of ELF1. Similarly, in osteosarcoma, miR‐214‐5p targets the ROCK1 3′UTR, and up‐regulation of miR‐214‐5p reduces ROCK1 protein expression, thereby inhibiting cell proliferation and migration.^46^ miR‐486‐3p directly targets DDR1 mRNA 3′‐UTR to inhibit DDR1 expression, decreasing cell proliferation and enhancing apoptosis in oral cancer.^47^

A large body of evidence suggests that mRNAs and non‐coding RNAs (like lncRNAs, pseudogenes, and circRNAs) can act as ceRNAs; they can competitively bind to microRNAs through miRNA response elements (MREs) and induce the development of human diseases including cancer.^48^, ^49^ We predicted that ELF1 and ZNRD1‐AS1 had the same MRE sequence: AGUCUUA (AGTCTTA), which competes to bind with miR‐499a‐5p. Therefore, ZNRD1‐AS1 can be considered as a ceRNA to compete with miR‐499a‐5p, to reduce its negative regulation of ELF1 and promote the formation of VM in gliomas. Similarly, FAM225A exerts a ceRNA effect and competes for binding to miR‐590‐3p and miR‐1275, thereby reducing their inhibitory effect on ITGβ3 and promoting proliferation and invasion of nasopharyngeal carcinoma cells.^50^ In gallbladder carcinoma, PVT1 competitively binds to miR‐143, reduces miR‐143's inhibitory effect on HK2 and promotes cell proliferation and migration.^51^

The increasement of EMI1 contributes to the development of human solid tumours and chromosomal instability.^52^ Our results revealed EMI1 expression increased in gliomas, and knockdown of EMI1 reduced cell VM formation. Similarly, EMI1 is abundant in squamous cell lung cancer tissues and cells, down‐regulating EMI1 promotes apoptosis, and EMI1 is positively correlated with tumorigenesis and poor prognoses of squamous cell lung cancers.^53^ Down‐regulation of EMI1 interferes with DNA synthesis in varieties of tumour, such as glioblastoma and breast cancer, inhibiting cancer cell growth and enhancing its sensitivity to chemotherapy and radiation therapy.^54^ JASPAR software predicted and confirmed by ChIP experiments that ELF1 bound to the EMI1 promoter region. Up‐regulation of ELF1 enhanced EMI1 mRNA and protein expression, whereas down‐regulation of ELF1 showed the opposite effects. A previous study indicates that in MCF‐7 breast cancer cells, ELF1 plays a transcriptional regulatory role, binding to the human Pygopus2 promoter region and activating its expression, promoting breast cancer cell growth.^55^ Our study confirmed that EMI1 was affected by ELF1 transcriptional regulation and was involved in the regulation of glioma cell VM mediated by ZRND1‐AS1/miR‐499a‐5p/ELF1.

In in vivo experiments, the overexpression of 144aa‐uORF and underexpression of ZNRD1‐AS1, ELF1 alone or in combination could significantly inhibit the xenograft volume in nude mice, prolong their survival time and reduce tumour VM density; the use of the combination allowed the nude mice to achieve the minimal xenograft volume, the longest survival time and the most sparse tumour VM density. The results showed that the combination of up‐regulation of 144aa‐uORF and down‐regulation of ZNRD1‐AS1, ELF1 had a potential application value.

We first proofed degradation of ZNRD1‐AS1 by low expression ZNRD1‐AS1‐144aa‐uORF was weakened though the NMD pathway, which increased the expression of ZNRD1‐AS1, overexpressed ZNRD1‐AS1 competitively bound to miR‐499a‐5p and reduced the negative regulation of ELF1 by miR‐499a‐5p. ELF1 expression was increased, and its transcriptional regulation activated EMI1 expression, promoting cell proliferation, migration, invasion and VM formation in gliomas. This study revealed a novel mechanism by which the ZNRD1‐AS1‐144aa‐uORF regulated vasculogenic mimicry in the gliomas via the ZNRD1‐AS1/miR‐499a‐5p/ELF1/EMI1 pathway, providing a new therapeutic strategy for gliomas.

## CONFLICT OF INTEREST

The authors declare no competing interests.

## AUTHOR CONTRIBUTIONS

YL contributed to the experimental design, manuscript draft and data analysis. MW contributed to the experimental implementation, manuscript draft and data analysis. YX designed the experiments. CY, XL, XR and SS performed the experiments. CH, JZ, DW and ZL analysed the data. MW conceived or designed the experiments, performed the experiments and wrote the manuscript. All authors read and approved the final manuscript.

## Supporting information

Table S1

Fig S1

## Data Availability

The data that support the findings of this study are available on request from the corresponding author. The data are not publicly available due to privacy or ethical restrictions.
