# EXPRESSION OF CONCERN: An Upstream Open Reading Frame Regulates Vasculogenic Mimicry of Glioma via ZNRD1‐AS1/miR‐499a‐5p/ELF1/EMI1 Pathway

**DOI:** 10.1111/jcmm.71268

**Published:** 2026-06-30

**EXPRESSION OF CONCERN**: M. Wang , C. Yang , X. Liu , J. Zheng , Y. Xue , X. Ruan , S. Shen , D. Wang , Z. Li , H. Cai and Y. Liu , “An Upstream Open Reading Frame Regulates Vasculogenic Mimicry of Glioma via ZNRD1‐AS1/miR‐499a‐5p/ELF1/EMI1 Pathway,” Journal of Cellular and Molecular Medicine 24, no. 11 (2020): 6120–6136, 10.1111/jcmm.15217.32368853 PMC7294115

This Expression of Concern is for the above article, published online on 05 May 2020 in Wiley Online Library (wileyonlinelibrary.com), and has been issued by agreement between the journal Editor‐in‐Chief, Stefan N. Constantinescu; the Foundation for Cellular and Molecular Medicine; and Wiley & Sons Ltd. The Expression of Concern has been agreed upon due to the identification of duplication between the U251 ZNRD1‐AS1(−) NC + miR‐499a‐5p(+) NC invasion panel in Figure 3I and the U87 control migration panel in Figure 5C. The authors did not respond to comment on the concerns despite repeated attempts to contact them. Therefore, the journal has decided to issue an Expression of Concern to inform and alert readers.

