# Supplementary material for: An upstream open reading frame regulates vasculogenic mimicry of glioma via ZNRD1‐AS1/miR‐499a‐5p/ELF1/EMI1 pathway
Source: J Cell Mol Med. 2020 May 5;24(11):6120–36. doi: 10.1111/jcmm.15217 (PMC7294115; doi:10.1111/jcmm.15217)
Supplement: Supplementary file 1 — Table S1 [file JCMM-24-6120-s001.doc]

| Primer or Probe | Gene | Sequence (5'->3') or Assay ID |
| --- | --- | --- |
| Primer | ZNRD1-AS1 | F: GAAGCTGGCATCCTCCTATGTCAC  R: TCACCAGAAGCAAGGAAGCACAC |
|  | UPF1 | F: GTCTTCCTCCTCGGCTTCAT  R: GCTGTCCCAGTTGATGTCCT |
|  | UPF2 | F:AAAATCTACAGCAACGAAGTGG  R:TCTTGTTAACATGACAAACGGC |
|  | SMG1 | F:GTACGGTGAATTAATCCAACCG  R:GTTTCAACTTGAGCAAACATGC |
|  | ELF1 | F:CCAAGTTCCAGTGGTTGTGTCTCC  R:GTACCTGCTGATGGATCTGTGCTG |
|  | EMI1 | F:GCCAGAGGAAATTTTAGACTGC  R:CCAAGTTGTGCTCACTTTAGAC |
|  | GAPDH | F: GGTGAAGGTCGGAGTCAACG  R: CCATGTAGTTGAGGTCAATGAAG |
| Probe | miR-499a-5p | 001352(Applied biosystems) |
|  | U6 | 001973(Applied biosystems) |

**Supplementary Table 1**. Primers and probes used for RT-qPCR.

One-Step SYBR PrimeScript RT-PCR cycling conditions were as follows: 5 minutes at 42°C, 10 seconds at 95°C, 40 cycles of 3 seconds at 95°C, and 30 seconds at 60°C.

The reverse transcription was set as follows: 30 minutes at 16°C, 30 minutes at 42°C, and 5 minutes at 85°C. PCR conditions were set as follows: 2 minutes at 50°C, 10 minutes at 95°C, 40 cycles of 15 seconds at 95°C and 1 minutes at 60°C.
