# Supplementary material for: An upstream open reading frame regulates vasculogenic mimicry of glioma via ZNRD1‐AS1/miR‐499a‐5p/ELF1/EMI1 pathway
Source: J Cell Mol Med. 2020 May 5;24(11):6120–36. doi: 10.1111/jcmm.15217 (PMC7294115; doi:10.1111/jcmm.15217)
Supplement: Supplementary file 2 — Fig S1 [file JCMM-24-6120-s002.doc]

**Supplementary Figure 1. miRNA microarrays data in U87 and U251 cells.**

**(A)** miRNA gene expression profiles as obtained from samples in three groups as indicated. **(B)** qRT-PCR was performed to validate the selected molecules. Data are presented as the mean ± SD (n=3 in each group). ******P* < 0.05,*******P* < 0.01 versus sh-NC group.
